# A sleeping phantom leg awakened following hemicolectomy, thrombosis, and chemotherapy: a case report

**DOI:** 10.1186/1752-1947-5-203

**Published:** 2011-05-25

**Authors:** Melita J Giummarra, John L Bradshaw, Michael ER Nicholls, Nellie Georgiou-Karistianis, Stephen J Gibson

**Affiliations:** 1 Experimental Neuropsychology Research Unit, School of Psychology and Psychiatry, Monash University, Clayton, Victoria 3800, Australia; 2 School of Psychology, Flinders University, Bedford Park 5042, South Australia, Australia; 3 National Ageing Research Institute, Parkville, Victoria 3052, Australia; 4 Caulfield General Medical Centre, Caulfield, Victoria 3162, Australia

## Abstract

**Introduction:**

We describe the case of a patient who experienced phantom pain that began 42 years after right above-the-knee amputation. Immediately prior to phantom pain onset, this long-term amputee had experienced, in rapid succession, cancer, hemicolectomy, chemotherapy, and thrombotic occlusion. Very little has been published to date on the association between chemotherapy and exacerbation of neuropathic pain in amputees, let alone the phenomenon of bringing about pain in amputees who have been pain-free for many decades. While this patient presented with a unique profile following a rare sequence of medical events, his case should be recognized considering the frequent co-occurrence of osteomyelitis, chemotherapy, and amputation.

**Case presentation:**

A 68-year-old Australian Caucasian man presented 42 years after right above-the-knee amputation with phantom pain immediately following hemicolectomy, thrombotic occlusion in the amputated leg, and chemotherapy treatment with leucovorin and 5-fluorouracil. He exhibited probable hyperalgesia with a reduced pinprick threshold and increased stump sensitivity, indicating likely peripheral and central sensitization.

**Conclusion:**

Our patient, who had long-term nerve injury due to amputation, together with recent ischemic nerve and tissue injury due to thrombosis, exhibited likely chemotherapy-induced neuropathy. While he presented with unique treatment needs, cases such as this one may actually be quite common considering that osteosarcoma can frequently lead to amputation and be followed by chemotherapy. The increased susceptibility of amputees to developing potentially intractable chemotherapy-induced neuropathic pain should be taken into consideration throughout the course of chemotherapy treatment. Patients in whom chronic phantom pain then develops, perhaps together with mobility issues, inevitably place greater demands on healthcare service providers that require treatment by various clinical specialists, including oncologists, neurologists, prosthetists, and, most frequently, general practitioners.

## Introduction

Phantom pain in amputees usually emerges immediately after limb loss and tends to become less troublesome with time [1]; however, some rare patients exhibit late-onset phantom pain [2,3]. The patient described in the present case report began to experience chronic stump and phantom pain 42 years after the original traumatic amputation, apparently triggered by later-occurring hemicolectomy, subsequent thrombotic occlusion in the amputated limb, and chemotherapy.

## Case presentation

Our patient was a 68-year-old Australian Caucasian man who had a right above-the-knee amputation following a motorcycle accident in 1959, when he was 19 years of age. He initially perceived a painless phantom that dissipated soon after amputation. He did not have painful neuromata, but experienced paroxysmal shock-like stump pain two to three times yearly that would settle within 24 hours. We first assessed our patient's phantom pain in a questionnaire study in 2005 [4], three years after the onset of his phantom pain, and more recently via an interview and clinical examination in 2009 conducted to investigate his late-onset phantom pain. The patient provided written, informed consent for the publication of this case report, and both studies were approved by local and hospital ethics committees.

In 2002, our patient was diagnosed with moderate to poorly differentiated adenocarcinoma which had infiltrated through the full thickness of the bowel wall and into one regional lymph node. He promptly underwent right hemicolectomy. Fifteen days later he was diagnosed with pulmonary emboli and secondary pneumonia. Thrombotic occlusion had developed in the right superficial femoral vein approximately 5 cm distal to the long saphenous junction and extending proximally to the level of the distal common femoral artery. The patient was advised against prosthesis use until the blood clot cleared approximately four months after the initial surgery.

Our patient completed a six-month course of chemotherapy with leucovorin 38 mg and 5-fluorouracil (5-FU) 800 mg, which were administered with domperidone 10 mg and dexamethasone 4 mg to 8 mg. There was no prophylactic administration of vitamin E before chemotherapy. Little note was made of the effect that these agents had on our patient's stump and phantom pain, except that he was advised to bandage his swollen stump during the third cycle and he reported nerve pain in the stump by the sixth cycle. The possible cause of stump swelling was not recorded.

Our patient noted the presence of a painful phantom foot, telescoped near the stump, and a definite increase in stump pain and hyperalgesia, which was particularly pronounced after prosthesis use, which began during the course of chemotherapy treatment. He presently takes carbamazepine (200 mg daily) and tramadol (200 mg daily) to manage his pain. Our patient is unable to differentiate between his stump and phantom pain, as they both occur within the same region, often simultaneously, and are characterized by the same sensations. Deep manipulation of the stump (with fingers) now triggers shock-type pains; however, providing even pressure with the prosthesis helps to alleviate pain, indicating the absence of any continuing irritation of the stump. The phantom sometimes feels cold, but never hot or burning.

Our patient's pain is exacerbated by sitting, increased levels of activity, heavy lifting, hot weather, sweating, and stress. He has never noticed any increase or change in pain in relation to toileting, having a full bladder or bowel, or genital stimulation. He finds that walking and keeping occupied reduces his pain. On the basis of the McGill Pain Questionnaire [5], he described his pain as jumping, tingling, aching, intense, numb, cold, and nagging (see Table 1 for pain intensity and unpleasantness ratings).

**Table 1:** Intensity and unpleasantness of stump and phantom pain in 2005 when the patient was first interviewed and at 2009 follow-up

Level of pain	2005	2009
Stump pain		
Intensity (constant)^a^	70	25
Intensity during episode of pain^a^	70	80
Unpleasantness^b^	70	35
Phantom pain		
Intensity (constant)^a^	60	30
Intensity during episode of pain^a^	70	80
Unpleasantness^b^	50	35

^a^Rated on a scale where 0 means no pain and 100 means the worst possible pain; ^b^rated on a scale where 0 means not unpleasant pain and 100 means intolerable pain.

On the Leeds assessment of neuropathic symptoms and signs pain scale [6], our patient scored 7 out of 16, responding positively to "having pain that feels like strange sensations in the skin characterised as pricking, tingling, or pins and needles" and "having pain that comes on suddenly in bursts for no apparent reason when he is still."

The patient did not exhibit allodynia on the stump when lightly stroked with cotton wool, but exhibited hyperalgesia and a reduced pinprick threshold in the stump region (pinprick was rated at 45 out of 100 on the Visual Analogue Scale (VAS), where a score of 0 is not painful and a score of 100 is the worst possible pain), compared to the arm (8 out of 100) and the lower shin of the intact leg (10 out of 100). The patient's perception threshold to Von Frey filaments was the same between his arm, stump, equivalent region on the intact leg, and lower shin on the intact leg at a pressure of 2.05 g, indicating diminished protective sensation in all regions. In the stump, 15.00 g was perceived as just painful (VAS score 15 out of 100). When tested for temporal summation (10 applications of the 15 g filament at a frequency of 1 second), the patient experienced marked wind-up, with an increase in pain intensity to 56 out of 100. Given the reduced protective sensations noted above, such a pattern may be considered suggestive of hyperpathia.

## Discussion

The patient described in the present case report experienced late-onset chronic stump and phantom pain after bowel surgery and chemotherapy with thrombotic occlusion in the amputated leg. He had presented with reduced pinprick threshold on his stump and diminished nerve function in all regions. Three mechanisms may have interacted to initiate and maintain his pain: (1) ischemia-induced neuropathy; (2) chemotherapy-induced peripheral neuropathy (CIPN), of which he was at greater risk considering his recent ischemic obstruction; and (3) central reorganization due to surgery and new peripheral nociceptive input from damaged nerves.

Denervation typically triggers reorganization of the sensory and motor maps of the denervated limb and is associated with phantom pain [7]. While remapping of the sensory homunculus occurs soon after amputation (for example, lower-limb amputation resulting in the foot representation's responding to stimulation of the upper leg or the genitals), over time these patterns can change. The hemicolectomy itself may potentially have influenced the leg central nervous system (CNS) representation, but this is unlikely because our patient's pain was not triggered or exacerbated by bladder or bowel functioning or by stimulation of "typical" homuncular regions such as the lower back or hip.

Thrombotic occlusion and ischemia can cause neuropathic complications, and vascular mechanisms such as decreased blood flow and cooler stump temperatures are associated with increased phantom pain [8]. Amputees with blood clot etiology experience exacerbated phantom pain and higher cutaneous pain thresholds, suggesting that thrombosis and associated nerve injury have a unique effect on pain generation and perception [9]. Patients with phantom pain exhibit greater sympathetic responses to personal stressors, with cardiovascular over-reactivity and increased heart rate and systolic blood pressure, which are also consistent with the circumstances in the present case, in which our patient experienced heightened pain during increased autonomic and emotional arousal. The triggers of our patient's phantom pain indicate possible autonomic nervous system involvement and warrant further investigation.

CIPN is experienced by up to 50% of cancer survivors and is more common among those with pre-existing peripheral neuropathy, such as amputation [10] or peripheral neuropathy [11], even when these patients are given "safe" treatment doses [12]. Degeneration of the peripheral nerves, particularly in patients with pre-existing neuropathy, may cause irreversible changes in pain gating through the dorsal and ventral horns, leading to altered central pain processing. While 5-FU, with which our patient was treated, is not typically identified as causing CIPN, there are at least two prior case reports of 5-FU-induced neuropathy [13,14]. Our patient presented with general diminished protection at all peripheral regions, possibly due to age-related degenerative processes or to the rare occurrence of 5-FU-induced sensorimotor axonal neuropathy.

The pain system changes dynamically in response to ongoing activation. Nerves severed by amputation or injured through CIPN or vascular occlusion generate high rates of ectopic activity, resulting in paroxysmal neuropathic pain [15], which is consistent with our patient's pain. He had increased pain sensitivity and excitability of the peripheral nerve fibers, particularly the A-fibers as indicated by punctate hyperalgesia [16], in the stump following chemotherapy. Damage to the peripheral nerves may have caused increased sensitivity of neurons in the dorsal horn and supra-spinal regions, resulting in central sensitization [17], eventuating in the perception of chronic phantom pain. The clinical examination also indicated hyperpathia in our patient, which is thought to be a CNS disorder following central deafferentation.

## Conclusions

In summary, in the present case, the patient experienced late-onset phantom pain 42 years following amputation. The rare combination of hemicolectomy, venous thrombosis, pulmonary emboli, anticoagulation, and chemotherapy with 5-FU and leucovorin likely caused a sequence of neuronal changes that resulted in the patient's perception of chronic and troublesome phantom and stump pain. This case highlights that even a previously modified CNS following amputation retains neuroplasticity in response to a new assault, with the capacity to awaken a sleeping phantom that is characterized by bothersome chronic pain. Indeed, our patient first experienced phantom pain many years after amputation, even though the initial injury did not result in such pain. Ultimately, these mechanisms must be considered in cancer treatment of amputees and patients with pre-existing neuropathy.

## Consent

Written informed consent was obtained from the patient for publication of this case report and any accompanying images. A copy of the written consent is available for review by the Editor-in-Chief of this journal.

## Competing interests

The authors declare that they have no competing interests.

## Authors' contributions

MG conducted the initial questionnaire study, followed up the patient's hospital-based medical records, conducted further interviews and sensory testing with the patient and was the principal author in writing and editing the manuscript. SG was involved in the initial questionnaire, provided guidance in exploring the etiology of the patient's pain and sensory testing protocols, and contributed to the writing and editing of the manuscript. JLB, MERN, and NGK were involved in the initial questionnaire, participated in discussions about the etiology of the patient's pain, and contributed to the writing and editing of the manuscript. All authors read and approved the final manuscript.
